# Supplementary material for: Integrative genomic analysis of adult mixed phenotype acute leukemia delineates lineage associated molecular subtypes
Source: Nat Commun. 2018 Jul 10;9:2670. doi: 10.1038/s41467-018-04924-z (PMC6039465; doi:10.1038/s41467-018-04924-z)
Supplement: Supplementary file 1 — Supplementary Information [file 41467_2018_4924_MOESM1_ESM.pdf]

Supplementary Table 1. The 295 genes targeted by next-generation sequencing in MPAL patients

**Gene name**									
ABCC9	*CALR*	*CUL5*	*FANCD2*	*HIST1H2BF*	*LEF1*	*NBN*	*PLA2G2D*	*SF3B1*	*TINF2 (TIN2)*
ABL1	*CARD11*	*CUX1*	*FANCE*	*HIST1H3D*	*LRP1B*	*NCOR1*	*PLCG2*	*SFRS1*	*TLR2*
ACTG1	*CBL*	*CYLD*	*FANCG*	*HIST1H4D*	*LTB*	*NCOR2*	*POT1*	*SFRS7*	*TLR9*
AKT1	*CBLB*	*DAXX*	*FANCI*	*HNRNPK*	*LUC7L2*	*NF1*	*POU2AF1*	*SGK1*	*TNFAIP3*
ANKRD11	*CCND1*	*DCLRE1C*	*FANCL*	*HRAS*	*LYN*	*NFE2*	*PRDM1*	*SH2B3*	*TNFRSF14*
ARID1A	*CCND3*	*DDX3X*	*FAS*	*ICOS*	*MALT1*	*NFKB1*	*PRKCB*	*SHH*	*TNKS*
ARID1B	*CD200*	*DIS3*	*FAT1*	*ID3*	*MAP2K1*	*NFKB2*	*PTEN*	*SMAD2*	*TOX*
ARID2	*CD274*	*DKC1*	*FAT3*	*IDH1*	*MAPK1*	*NFKBIA*	*PTPN1*	*SMC1A*	*TP53*
ARID5B	*CD58*	*DLC1*	*FBXW7*	*IDH2*	*MAX*	*NFKBIE*	*PTPN11*	*SMC3*	*TRAF3*
ARPP21	*CD79A*	*DNM2*	*FGFR3*	*IKBKA*	*MDM2*	*NOTCH1*	*RAD21*	*SMC5*	*TRAF6*
ASXL1	*CD79B*	*DNMT1*	*FLI1*	*IKZF1*	*MED12*	*NOTCH2*	*RAD51C*	*SNX7*	*TYK2*
ATF7IP	*CDK4*	*DNMT3A*	*FLT3*	*IKZF2*	*MEF2B*	*NPM1*	*RAG1*	*SOCS1*	*TYK3*
ATM	*CDKN2A*	*DNMT3B*	*FNDC3A*	*IKZF3*	*MEF2C*	*NR3C2*	*RAG2*	*SOX5*	*U2AF1*
ATRX	*CDKN2B*	*EBF1*	*FOXP1*	*IL7R*	*MGA*	*NRAS*	*RASA2*	*SP140*	*U2AF2*
B2M	*CDKN2C*	*ECT2L*	*FYN*	*IRAK1*	*miR125a*	*NSD2*	*RB1*	*SPEN*	*UBR5*
BCL10	*CEBPA*	*EED*	*G6PC3*	*IRAK4*	*miR-142*	*NT5C2*	*REL*	*SPIB*	*USP29*
BCL2	*CEBPE*	*EGR1*	*GAB2*	*IRF1*	*mIR155*	*PAG1*	*RELA*	*SRSF2*	*VPREB1*
BCL6	*CHD2*	*EGR2*	*GATA1*	*IRF4*	*mIR15a*	*PALB2*	*RELB*	*STAG1*	*WHSC1*
BCL7A	*CHK2*	*ELANE*	*GATA2*	*IRF7*	*mIR16-1*	*PAX5*	*RELN*	*STAG2*	*WHSC1L1*
BCOR	*CIITA*	*EP300*	*GATA3*	*ITPKB*	*MIR17HG*	*PDCD1*	*RHOA*	*STAT1*	*WT1*
BCR	*CNOT3*	*EPHA7*	*GCET2*	*JAK1*	*mIR21*	*PDCD1LG2*	*RIPK1*	*STAT3*	*XPO1*
BIRC3	*CREBBP*	*EPOR*	*GFI1B*	*JAK2*	*mir34b*	*PDGFRB*	*ROBO1*	*SUZ12*	*ZAP70*
BLK	*CRLF2*	*ERG*	*GNA13*	*JAK3*	*mir34c*	*PEG3*	*ROR1*	*SYK*	*ZMYM2*
BMI1	*CSF2RA*	*ETV6*	*GNAS*	*JARID2*	*MLL*	*PHF6*	*RPL10*	*TBL1XR1*	*ZMYM3*
BRAF	*CSF3R*	*EZH2*	*GNB1*	*KDM4C*	*MLL2*	*PHIP*	*RPL5*	*TCF3*	*ZRSR2*
BRIP1	*CTBP1*	*FAM46C*	*GPRC5A*	*KDM6A*	*MLL3*	*PIGA*	*RUNX1*	*TERC*	
BTG1	*CTBP2*	*FAM5C*	*HAX1*	*KIT*	*MPL*	*PIK3CA*	*RUNX2*	*TERT*	
BTK	*CTCF*	*FANCA*	*HIST1H1E*	*KLHL6*	*MS4A1*	*PIK3CB*	*SAMHD1*	*TET1*	
BTLA	*CTLA4*	*FANCB*	*HIST1H2AD*	*KRAS*	*MYB*	*PIK3CG*	*SETBP1*	*TET2*	
C22orf194	*CTNNA1*	*FANCC*	*HIST1H2BE*	*LAMB4*	*MYD88*	*PIK3R1*	*SETD2*	*TGDS*	

Supplementary Table 2. Summary of high-confidence somatic mutations detected in 31 MPAL samples.

UPN	Immunosubtype	Function	Gene	Exonic Function	Transcript ID	Nucleotide change	AA change	VAF
MDA030	Myeloid/B	exonic	*ASXL1*	stopgain SNV	uc021wbw.1	c.G3110A	p.W1037X	0.25
MDA017	Myeloid/B	exonic	*ASXL1*	stopgain SNV	uc021wbw.1	c.C2083T	p.Q695X	0.20815
MDA007	Myeloid/B	exonic	*ASXL1*	stopgain SNV	uc021wbw.1	c.C1534T	p.Q512X	0.429336
MDA029	Myeloid/T	exonic	*ASXL1*	frameshift insertion	uc021wbw.1	c.1926_1927insG	p.G642fs	0.1335
MDA014	Myeloid/T	exonic	*ASXL1*	frameshift insertion	uc021wbw.1	c.1926_1927insG	p.G642fs	0.1635
MDA028	Myeloid/B	exonic	*BCOR*	stopgain SNV	uc004dem.4	c.C1288T	p.Q430X	0.258985
MDA003	Myeloid/T	exonic	*BRCA2*	stopgain SNV	uc001uub.1	c.G10024T	p.E3342X	0.507415
MDA015	Myeloid/T	exonic	*CBL*	frameshift deletion	uc001pwe.3	c.1226_1227del	p.409_409del	0.7266
MDA013	Myeloid/T	exonic	*CCND3*	frameshift insertion	uc003orn.3	c.820_821insGGCCCCCCGGGGCTCC	p.S274fs	0.2621
MDA008	Myeloid/T	exonic	*CDKN2A*	stopgain SNV	uc003zpk.3	c.C238T	p.R80X	0.977124
MDA020	Myeloid/T	exonic	*CEBPA*	nonframeshift deletion	uc002nun.3	c.308_313del	p.103_105del	0.3065
MDA004	Myeloid/T	exonic	*CNOT3*	stopgain SNV	uc002qdk.2	c.C627A	p.Y209X	0.345499
MDA014	Myeloid/T	exonic	*CSF3R*	nonsynonymous SNV	uc001cav.2	c.C1853T	p.T618I	0.417695
MDA012	Myeloid/B	exonic	*DNMT3A*	nonsynonymous SNV	uc002rgc.3	c.G1627T	p.G543C	0.470657
MDA009	Myeloid/T	exonic	*DNMT3A*	stopgain SNV	uc002rgc.3	c.C2311T	p.R771X	0.838912
MDA005	Myeloid/T	exonic	*DNMT3A*	stopgain SNV	uc002rgc.3	c.C2311T	p.R771X	0.441137
MDA001	Myeloid/T	splicing	*DNMT3A*					0.407725
MDA026	Myeloid/T	exonic	*DNMT3A*	frameshift deletion	uc002rgc.3	c.2069delT	p.V690fs	0.8387
MDA024	Myeloid/T	exonic	*DNMT3A*	frameshift deletion	uc002rgc.3	c.2443delC	p.L815fs	0.8807
MDA023	Myeloid/T	exonic	*DNMT3A*	frameshift deletion	uc002rgc.3	c.2296_2297del	p.766_766del	0.7181
MDA016	Myeloid/B	exonic	*EGFR*	nonsynonymous SNV	uc003tqk.3	c.C2543T	p.P848L	0.582251
MDA029	Myeloid/T	exonic	*ETV6*	nonframeshift insertion	uc001qzz.3	c.1031_1032insCCCCCC	p.Y344delinsYPP	0.12
MDA029	Myeloid/T	exonic	*ETV6*	nonframeshift insertion	uc001qzz.3	c.1247_1248insGGC	p.L416delinsLA	0.0544
MDA024	Myeloid/T	exonic	*ETV6*	frameshift insertion	uc001qzz.3	c.562_563insCTGG	p.T188fs	0.204
MDA024	Myeloid/T	exonic	*ETV6*	nonframeshift insertion	uc001qzz.3	c.1159_1160insCAAGGTCAGGTC	p.T387delinsTRSGP	0.1854
MDA027	Myeloid/T	exonic	*EZH2*	nonsynonymous SNV	uc003wfb.2	c.T2079A	p.N693K	0.308231
MDA004	Myeloid/T	exonic	*FBXW7*	nonsynonymous SNV	uc003ims.3	c.C1393T	p.R465C	0.332203
MDA027	Myeloid/T	exonic	*FLT3*	nonsynonymous SNV	uc001urw.3	c.G2503T	p.D835Y	0.24086
MDA018	Myeloid/B	exonic	*FLT3*	nonsynonymous SNV	uc001urw.3	c.G2503T	p.D835Y	0.053493
MDA021	Myeloid/B	exonic	*FLT3*	nonframeshift insertion	uc001urw.3	c.1800_1801insCCCTTTCGTTGATTTCAGAGAATATGAATATGA	p.D600delinsDPFVDFREYEYD	0.1343
MDA012	Myeloid/B	exonic	*FLT3*	nonframeshift insertion	uc001urw.3	c.1784_1785insTTCCCAAGACCGCCCATCTCA	p.R595delinsIPKTAHLR	0.0963
MDA008	Myeloid/T	exonic	*FLT3*	nonframeshift insertion	uc001urw.3	c.1800_1801insCCCTAGGGTACAGGTGACCGGCTCCTCAGATAATGAGTACTT	p.D600delinsDPRVQVTGSSDNEYFYVDFREYEYD	0.2432

						CTACGTTGATTTCAGAGAATATGAATATGA		
MDA004	Myeloid/T	exonic	*GATA3*	nonsynonymous SNV	uc001ijz.3	c.G827A	p.R276Q	0.36385
MDA004	Myeloid/T	exonic	*GATA3*	frameshift insertion	uc001ijz.3	c.481_482insGCTTC	p.V161fs	0.2636
MDA022	Myeloid/B	exonic	*IDH1*	nonsynonymous SNV	uc002vcs.3	c.G395A	p.R132H	0.123864
MDA007	Myeloid/B	exonic	*IDH1*	nonsynonymous SNV	uc002vcs.3	c.C394A	p.R132S	0.221865
MDA026	Myeloid/T	exonic	*IDH2*	nonsynonymous SNV	uc002box.3	c.G419A	p.R140Q	0.46871
MDA024	Myeloid/T	exonic	*IDH2*	nonsynonymous SNV	uc002box.3	c.G419A	p.R140Q	0.428973
MDA021	Myeloid/B	exonic	*IDH2*	nonsynonymous SNV	uc002box.3	c.G419A	p.R140Q	0.465517
MDA014	Myeloid/T	exonic	*IDH2*	nonsynonymous SNV	uc002box.3	c.G419A	p.R140Q	0.441048
MDA009	Myeloid/T	exonic	*IDH2*	nonsynonymous SNV	uc002box.3	c.G419A	p.R140Q	0.422427
MDA005	Myeloid/T	exonic	*IDH2*	nonsynonymous SNV	uc002box.3	c.G419A	p.R140Q	0.454048
MDA001	Myeloid/T	exonic	*IDH2*	nonsynonymous SNV	uc002box.3	c.G419A	p.R140Q	0.33389
MDA015	Myeloid/T	exonic	*IKZF1*	stopgain SNV	uc003tow.4	c.C1305G	p.Y435X	0.911364
MDA001	Myeloid/T	exonic	*IKZF1*	stopgain SNV	uc003tow.4	c.C184T	p.Q62X	0.167573
MDA002	Myeloid/T	exonic	*IKZF2*	stopgain SNV	uc002vem.3	c.A391T	p.K131X	0.61869
MDA029	Myeloid/T	exonic	*IL7R*	nonframeshift insertion	uc003jjs.3	c.623_624insCCCTGATCACTATTTGTC	p.I208delinsIPDHYLS	0.0522
MDA029	Myeloid/T	exonic	*IL7R*	nonframeshift insertion	uc003jjs.3	c.730_731insGGCCCTCGGAATGCC	p.T244delinsRPSECP	0.0837
MDA006	Myeloid/T	exonic	*IL7R*	nonframeshift insertion	uc003jjs.3	c.726_727insCCCTGC	p.L242delinsLPC	0.3296
MDA004	Myeloid/T	exonic	*IL7R*	nonframeshift insertion	uc003jjs.3	c.732_733insGGCTGTTGGATA	p.T244delinsTGCWI	0.261
MDA024	Myeloid/T	exonic	*KRAS*	nonsynonymous SNV	uc001rgp.1	c.C437T	p.A146V	0.459893
MDA023	Myeloid/T	exonic	*KRAS*	nonsynonymous SNV	uc001rgp.1	c.C173T	p.T58I	0.462845
MDA009	Myeloid/T	exonic	*KRAS*	nonsynonymous SNV	uc001rgp.1	c.A182T	p.Q61L	0.451509
MDA001	Myeloid/T	splicing	*MED12*					0.099338
MDA020	Myeloid/T	exonic	*MLL2*	frameshift insertion	uc001rta.4	c.1125_1126insC	p.P375fs	0.1876
MDA013	Myeloid/T	exonic	*NF1*	stopgain SNV	uc002hge.2	c.C1381T	p.R461X	0.828571
MDA011	Myeloid/B	exonic;splicing	*NF1;NF1*	nonsynonymous SNV	uc002hgg.3	c.G6642C	p.E2214D	0.108949
MDA026	Myeloid/T	exonic	*NOTCH1*	nonsynonymous SNV	uc004chz.3	c.T5033A	p.L1678Q	0.439863
MDA015	Myeloid/T	exonic	*NOTCH1*	nonsynonymous SNV	uc004chz.3	c.T4754C	p.L1585P	0.431579
MDA009	Myeloid/T	exonic	*NOTCH1*	nonsynonymous SNV	uc004chz.3	c.T4721C	p.L1574P	0.079365
MDA004	Myeloid/T	exonic	*NOTCH1*	nonsynonymous SNV	uc004chz.3	c.T4787A	p.L1596H	0.3
MDA029	Myeloid/T	exonic	*NOTCH1*	frameshift deletion	uc004chz.3	c.7541_7542del	p.2514_2514del	0.2773
MDA023	Myeloid/T	exonic	*NOTCH1*	nonframeshift insertion	uc004chz.3	c.4747_4748insCCG	p.E1583delinsPE	0.1587
MDA014	Myeloid/T	exonic	*NOTCH1*	nonframeshift insertion	uc004chz.3	c.5080_5081insTTA	p.Q1694delinsLQ	0.2108
MDA009	Myeloid/T	exonic	*NOTCH1*	frameshift deletion	uc004chz.3	c.7541_7542del	p.2514_2514del	0.3108
MDA006	Myeloid/T	exonic	*NOTCH1*	frameshift insertion	uc004chz.3	c.7327_7328insCC	p.V2443fs	0.25
MDA001	Myeloid/T	exonic	*NOTCH1*	nonframeshift insertion	uc004chz.3	c.4815_4816insGGT	p.V1605delinsVV	0.119

MDA026	Myeloid/T	exonic	*NRAS*	nonsynonymous SNV	uc009wgu.3	c.G35A	p.G12D	0.435654
MDA017	Myeloid/B	exonic	*NRAS*	nonsynonymous SNV	uc009wgu.3	c.G35A	p.G12D	0.176346
MDA016	Myeloid/B	exonic	*NRAS*	nonsynonymous SNV	uc009wgu.3	c.G34A	p.G12S	0.146008
MDA014	Myeloid/T	exonic	*NRAS*	nonsynonymous SNV	uc009wgu.3	c.G34C	p.G12R	0.386752
MDA012	Myeloid/B	exonic	*NRAS*	nonsynonymous SNV	uc009wgu.3	c.G35A	p.G12D	0.107296
MDA005	Myeloid/T	exonic	*NRAS*	nonsynonymous SNV	uc009wgu.3	c.G38A	p.G13D	0.409819
MDA002	Myeloid/T	exonic	*PHIP*	stopgain SNV	uc003pir.3	c.C181T	p.Q61X	0.252441
MDA016	Myeloid/B	exonic	*PTPN11*	nonsynonymous SNV	uc001ttw.1	c.A182T	p.D61V	0.413304
MDA021	Myeloid/B	exonic	*RUNX1*	nonsynonymous SNV	uc002yuk.4	c.G497A	p.R166Q	0.89128
MDA017	Myeloid/B	exonic	*RUNX1*	stopgain SNV	uc002yuk.4	c.C496T	p.R166X	0.298295
MDA012	Myeloid/B	exonic	*RUNX1*	nonsynonymous SNV	uc002yuk.4	c.A593G	p.D198G	0.483407
MDA010	Myeloid/B	splicing	*RUNX1*					0.315
MDA028	Myeloid/B	exonic	*RUNX1*	frameshift deletion	uc002yuk.4	c.950_953del	p.317_318del	0.2905
MDA027	Myeloid/T	exonic	*RUNX1*	frameshift insertion	uc002yuk.4	c.424_425insCC	p.A142fs	0.0615
MDA007	Myeloid/B	exonic	*RUNX1*	frameshift insertion	uc002yuk.4	c.997_998insGCGAC	p.P333fs	0.1875
MDA004	Myeloid/T	exonic	*RUNX1*	nonframeshift insertion	uc002yuk.4	c.493_494insTCG	p.G165delinsSG	0.2638
MDA017	Myeloid/B	exonic	*SETBP1*	nonsynonymous SNV	uc010dni.3	c.G2608A	p.G870S	0.223224
MDA017	Myeloid/B	exonic	*SF3B1*	nonsynonymous SNV	uc002uue.3	c.A2098G	p.K700E	0.212882
MDA029	Myeloid/T	exonic	*SH2B3*	nonsynonymous SNV	uc001tsf.3	c.G658A	p.G220R	0.648148
MDA004	Myeloid/T	exonic	*SH2B3*	nonsynonymous SNV	uc001tsf.3	c.T905C	p.L302P	0.382275
MDA004	Myeloid/T	exonic	*SH2B3*	nonsynonymous SNV	uc001tsf.3	c.C1098A	p.F366L	0.378685
MDA025	Myeloid/T	exonic	*SRSF2*	nonsynonymous SNV	uc002jsv.3	c.C284G	p.P95R	0.444717
MDA007	Myeloid/B	exonic	*SRSF2*	nonsynonymous SNV	uc002jsv.3	c.C284A	p.P95H	0.439469
MDA021	Myeloid/B	exonic	*SRSF2*	nonframeshift deletion	uc002jsv.3	c.284_307del	p.95_103del	0.3197
MDA012	Myeloid/B	exonic	*SRSF2*	nonframeshift insertion	uc002jsv.3	c.284_285insGCC	p.P95delinsRP	0.2699
MDA014	Myeloid/T	exonic	*STAG2*	stopgain SNV	uc004etz.4	c.C436T	p.R146X	0.891608
MDA005	Myeloid/T	exonic	*TCF3*	stopgain SNV	uc002ltt.4	c.C1111T	p.R371X	0.445748
MDA028	Myeloid/B	exonic	*TET2*	stopgain SNV	uc011cez.2	c.C1955G	p.S652X	0.307135
MDA012	Myeloid/B	exonic	*TET2*	stopgain SNV	uc011cez.2	c.A5794T	p.K1932X	0.497354
MDA003	Myeloid/T	splicing	*TP53*					0.771654
MDA011	Myeloid/B	exonic	*TP53*	frameshift deletion	uc002gih.3	c.988delC	p.L330fs	0.2174
MDA003	Myeloid/T	exonic	*TP53*	stopgain SNV	uc002gig.1	c.736delA	p.M246X	0.0848
MDA022	Myeloid/B	exonic	*WT1*	stopgain SNV	uc001mtn.2	c.C1142A	p.S381X	0.19526

Supplementary Table 3. Comparison of clinical characteristics between MPAL patients eligible for the study (N = 31) and removed from the study (N = 24).

	MPAL kept for the study	MPAL removed from the study	P - value
	N = 31	N = 24	
Median age, years (IQR)	53 (30-61.5)	44.5 (32-57.25)	0.415
**The # of prior therapy (%)**			NA
0	31 (100)	9 (38)*	
1	0	9 (38)	
2	0	3 (13)	
3	0	1 (4)	
4	0	1 (4)	
5	0	1 (4)	
**Immunophenotype (%)**			0.45
Myeloid-B	13 (42)	8 (33)	
Myeloid-T	18 (58)	15 (63)	
T-B	0	1 (4)	
Median WBC, x10^3/μL (IQR)	7 (3.1-18.2)	13.7 (1-83.45)	0.916
Median HGB, g/dL (IQR)	8.9 (8.25-10.9)	8.4 (8.05-9.9)	0.201
Median PLT, x10^3/μL (IQR)	56 (29-129.5)	61 (20.5-148.5)	0.824
Median PB blast percentage (IQR)	42 (12-70)	59.5 (6-94.75)	0.601
Median BM blast percentage (IQR)	78 (60.5-87)	65 (36-86)	0.126
Median LDH, IU/L (IQR)	831 (656-1120.5)	727.5 (435-1659.25)	0.627
**Cytogenetic abnormalities**			0.684
Normal	10	5	
Ph+	4	2	
11q23 rearrangement	1	2	
Complex	8	9	
Other	8	5	

*These patients were removed because baseline samples were not available or patient did not consent for the sample storage protocol. All patients who received prior therapy before the presentation to our institution were also not considered eligible for the study.
IQR, interquartile range; WBC, white blood cells; HGB, hemoglobin; PLT, platelets; BM, bone marrow; PB, peripheral blood; LDH, lactate dehydrogenase; Ph+, Philadelphia chromosome positive;

Supplementary Table 4. Clinical information including treatment history and response to therapy.

UID	Age	Subtype	CG	Initial treatment	Treatment Category	Methylation Class	Methylation/ Therapy Match	Best response	Cycles to achieve best response	Relapse	SCT	Status at last follow up	Breif clinical course
MPAL1	61	Myeloid/T	NK	HyperCVAD	ALL_type	ALL	Matched	CR	1		Haplo	Alive	Achieved CR with 1 cycle of HyperCVAD. Received total 4 cycles and underwent haploidentical SCT. Still alive at LFU.
MPAL2	41	Myeloid/T	Other	HyperCVAD	ALL_type	AML	Unmatched	NR				Dead	Refractory to the induction. Received 2nd cycle but lost follow up.
MPAL3	72	Myeloid/T	Complex	Cladribine + Cytarabine	AML_type	AML	Matched	NR				Dead	Received 4 additional cycles of decitabine and 1 cycle of evofosfamide but was refractory. Eventually died from multiple infections (pneumonia and disseminated HSV infection)
MPAL4	23	Myeloid/T	Other	CIA	AML_type	ALL	Unmatched	CR_i	1	Yes	MUD	Dead	Received 2 cycles and achieved CR_i and underwent for MUD SCT. Relapsed at Day 74.
MPAL5	45	Myeloid/T	NK	HyperCVAD	ALL_type	ALL	Matched	CR	1			Dead	Died of cerebral hemorrhage while in CR.
MPAL6	32	Myeloid/T	11q23	HyperCVAD	ALL_type	ALL	Matched	CR	1		Cord	Alive	Achieved CR with HyperCVAD and received total 7 cycles. Underwent for double-cord SCT and still alive at LFU.
MPAL7	86	Myeloid/B	Complex	None	NA	AML		Not evaluable				Alive	Did not receive therapy at our institution. Lost follow up.
MPAL8	25	Myeloid/T	Other	HyperCVAD	ALL_type	ALL	Matched	CR	1	Yes		Dead	Achieved CR with HyperCVAD and received total 4 cylces. Relapsed 9 months from initial CR. Salvage therapies with augmented hyperCVAD, MDM2 inhibitor and fludarabine+iarubicin+cytarabine+sorafenib but no response. Died of disseminated fungal infection.
MPAL9	30	Myeloid/T	Complex	HyperCVAD	ALL_type	ALL	Matched	CR_i	1		Cord	Alive	Achieved CR_i with HyperCVAD and received 1 cycle of consolidation. Underwent for double-cord SCT. Still alive at LFU.
MPAL10	9	Myeloid/B	Ph+	Cytarabine, vincristine, daunorubicine, prednisone, pegasparaginase	ALL_type	AML	Unmatched	CR_i	1		MUD	Alive	Achieved CR after the induction and underwent for MUD SCT. Alive at LFU.
MPAL11	23	Myeloid/B	Complex	HyperCVAD	ALL_type	ALL	Matched	CR	1		MUD	Alive	Achieved CR after HyperCVAD and received total 4 cycles. Underwent for MUD SCT and still laive at LFU.
MPAL12	48	Myeloid/B	Other	IA+vorinostat	AML_type	AML	Matched	CR_i	2	Yes	MUD	Dead	Achieved CR_i with IA+vorinostat and received 1 more cycle. Then underwent for MUD SCT but relapsed at 108 days. Further salvage therapies with ponatinib, quizartinib, TG02, fludarabine+cytarabine, evofosfamide, CIA, guadecitabine, SAR103168 and MEK inhibitor but no response. Died of sepsis.
MPAL13	28	Myeloid/T	NK	HyperCVAD	ALL_type	ALL	Matched	NR				Dead	Refractory to 2 cylces of HyperCVAD. Then recived nelarabine and Clofarabine/cyclophospamide but continued to be refractory. Died of unknown cause.
MPAL14	82	Myeloid/T	NK	Hydroxyurea	Other	ALL		Not evaluable				Dead	Only received hydroxyurea and went home. Cause of death unknown.
MPAL15	36	Myeloid/T	Complex	IA	AML_type	ALL	Unmatched	CR_i	1	Yes	MRD	Dead	Relapsed day 30 post transplant and received clofarabine, cytarabine, vincristine and dexamethasone and no response. Then high dose methotrexate and cytarabine but no response. Died of disease progression and GvHD.
MPAL16	18	Myeloid/B	Other	HyperCVAD	ALL_type	ALL	Matched	CR	1	Yes		Alive	Received 8 cycles of HyperCVAD but realpsed after 1.5 years. Then treated with augmented HyperCVAD and achieved CR2. Completed 8 cycles of this therapy and maintained remission until the LFU.
MPAL17	76	Myeloid/B	Complex	Clofarabine, cytarabine	AML_type	AML	Matched	CR	1	Yes		Dead	Short CR duration of 2 months and received decitabine + vorinostat for 2 cycles. No response. Went home and lost follow up.
MPAL18	77	Myeloid/B	Complex	Quizartinib	Other	ALL		Not evaluable				Dead	Briefly treated with quizartinib but died early with renal failure and pneumonia.
MPAL19	59	Myeloid/B	Ph+	HyperCVAD+dasatinib	ALL_type	AML	Unmatched	CR	1	Yes		Dead	Achieved CR after 1 cycle. 2nd cycle was complicated with subdural hematoma. From third cycle, took imatinib + vincristine and then switched to nilotinib only. Relapsed after a year and died of pneumonia MOF
MPAL20	30	Myeloid/T	Other	IA	AML_type	ALL	Unmatched	CR_i	1			Alive	Achieved CR_i afte 1 cycle. Went back to home country and lost follow up.
MPAL21	62	Myeloid/B	NK	None	NA	AML		Not evaluable				Dead	Consultation visit only and went home. Lost follow up.
MPAL22	59	Myeloid/B	NK	FLAG	AML_type	AML	Matched	CR_i	1	Yes		Dead	Received 6 cycles of consolidation with mitoxantrone/etoposide but relapsed 6 months later. Then received azacitidine, barasertib, fludarabine+cytarabine+gemtuzumab ozogamicin, and decitabine. Died of unknown cause.
MPAL23	47	Myeloid/T	Other	HyperCVAD	ALL_type	ALL	Matched	CR	1		MRD	Dead	Died of SCT complication. Respiratory failure.
MPAL24	60	Myeloid/T	Other	HyperCVAD	ALL_type	ALL	Matched	CR	1			Dead	Received consolidation for 4 cycles. Declined transplant. Cuase of death unknown.
MPAL25	70	Myeloid/T	NK	HyperCVAD	ALL_type	AML	Unmatched	NR				Dead	No response. Received idarubicine and cytarabine as salvage but lost follow up. Response to 2nd cycle unknown.
MPAL26	73	Myeloid/T	NK	Cloretazine	Other	ALL	Unmatched	CR_i	1			Dead	Achieved CR_i but died of pneumonia. Respiratory failure

MPAL27	60	Myeloid/T	NK	Clofarabine, cytarabine	AML_type	AML	Matched	CR	1	Yes		Dead	11 months of remission but relapsed. Subsequent salvage with BAY43-9006 with no response. Then IA+vorinostat and achieved CR2. Maintained CR2 for 6M but relapsed again. Then treated with augmented HyperCVADx 6 cycles and achieved CR3. Died of sepsis.
MPAL28	58	Myeloid/B	NK	HyperCVAD	ALL_type	ALL	Matched	CR	1	Yes		Dead	Received 7 cycles of HyperCVAD but relapsed 1 year after. Treated with IA, vincristine and dexamethasone and achieved CR_i again but died of pneumonia.
MPAL29	16	Myeloid/T	Complex	HyperCVAD	ALL_type	AML	Unmatched	CR	1	Yes	Cord/Haplo	Dead	Received 5 cycles of HyperCVAD and maintenance therapy for 30 cycles. Maintained CR for 7 years but relapsed. Again treated with HyperCVAD for 3 cylces and achieved CR2. Underwent for double cord SCT but 1 year after the transpalnt, relapsed again. Treated with fludarabine, cyclophosphamide, lenalidomide and NK cell infusion and underwent for haploidentical SCT but died of SCT complication.
MPAL30	60	Myeloid/B	Ph+	HyperCVAD+imatinib	ALL_type	ALL	Matched	CR	1			Dead	Went home and completed chemo but lost follow up.
MPAL31	53	Myeloid/B	Ph+	FIA	AML_type	AML	Matched	CR	1	Yes		Dead	Achieved CR with FIA and continued consolidation with dasatinib but relapsed 1.5 years after and then treated with HyperCVAD+dasatainib x 2 cycles with no response. Went to hospice.

UID: Unique Identifier, CG: cytogenetics, SCT: stem cell transplant, HyperCVAD: Hyperfractionated cyclophophamide, vincristine, doxorubicin, and dexamethason alternating with high dose methotrexate and cytarabine. CR: complete remission, CR_i: CR with incomplete count recovery, NR: no response, MUD: matched unrelated donor, MRD: matched related donor, IA: idarubicin and cytarabine, CIA: clofarabine, idarubicin and cytarabine, FLAG: fludarabine, cytarabine, and G-CSF, LFU: last follow-up.

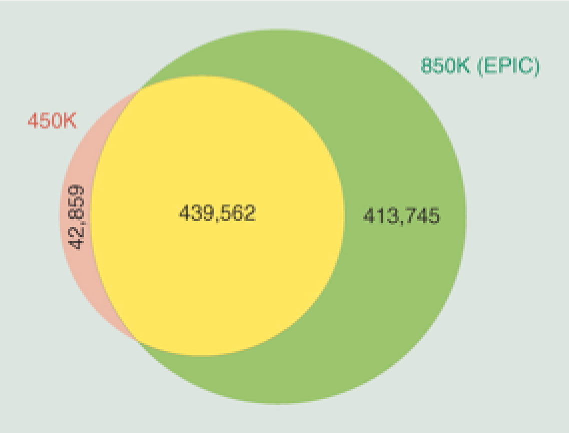

Supplementary Figure 1. Venn diagram showing overlapped and non-overlapped CpG probes between Illumina's HM450K platform and EPIC platform.

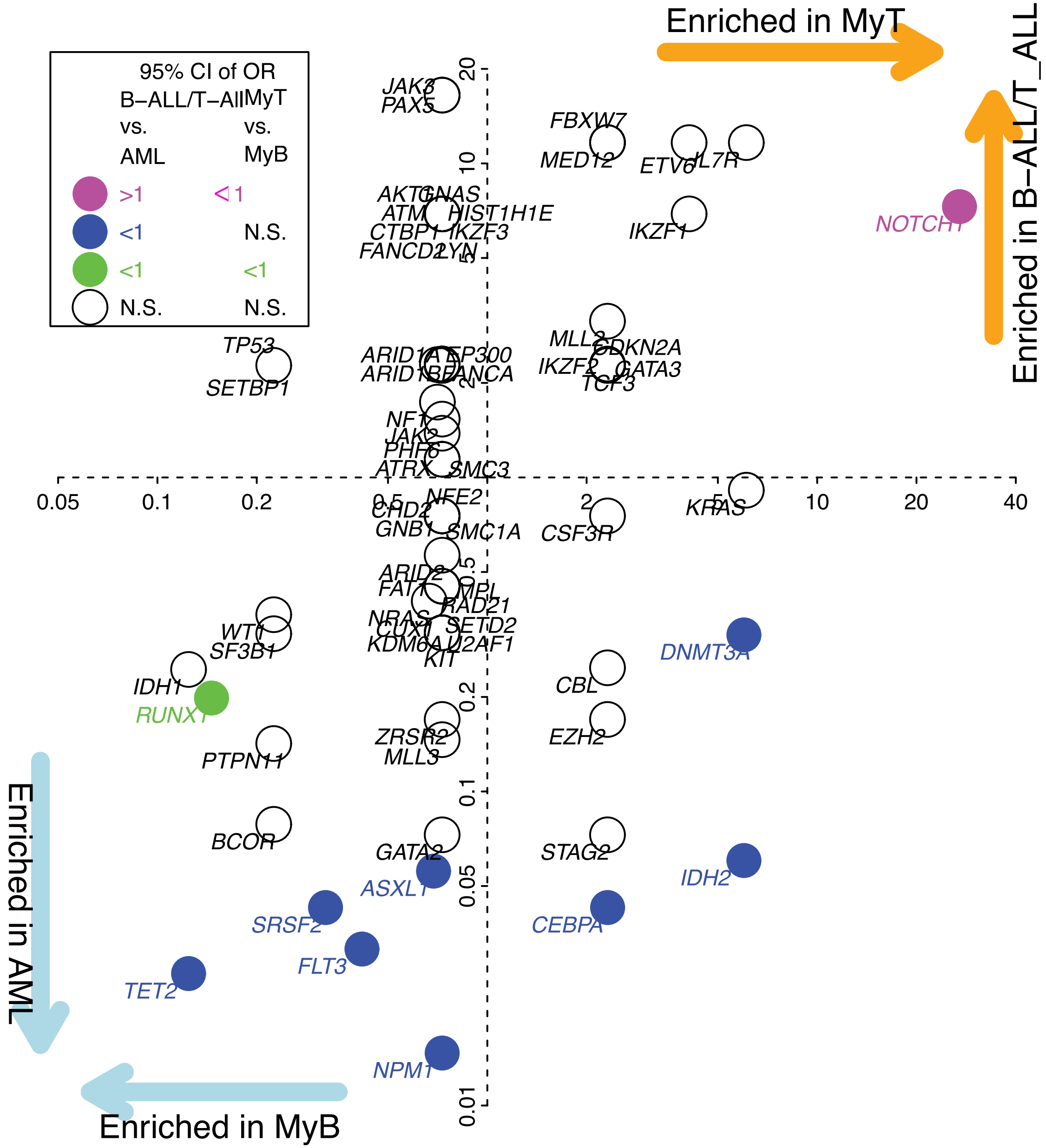

Supplementary Figure 2: Relative enrichment of each mutation against leukemia subtypes. X axis shows enrichment against MPAL (myeloid-B vs. myeloid-T) while Y axis shows enrichment against AML vs. ALL (either B-ALL or T-ALL). Colored dots represent statistical significance of enrichment to particulr subtype.

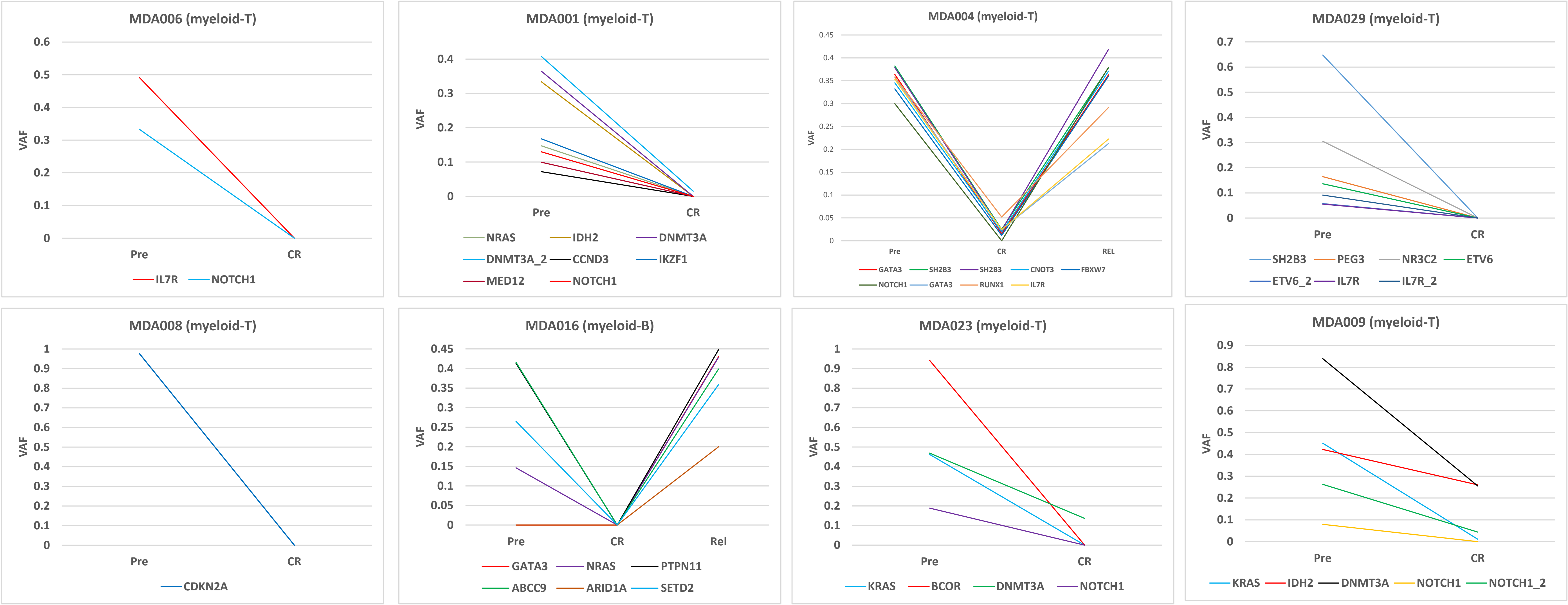

Supplementary Figure 3. Longitudinal sequencing of 8 MPAL cases at pre-treatment and complete remission (CR). Three cases had DNMT3A mutations and all the mutations persisted at CR. Relapsed samples were sequenced in 2 cases (MDA004 and MDA016). In MDA016, we detected acquisition of *GATA3* and *ARID1A* mutations. This case also lost MPO positivity at relapse which was positive in 48% cells at diagnosis.

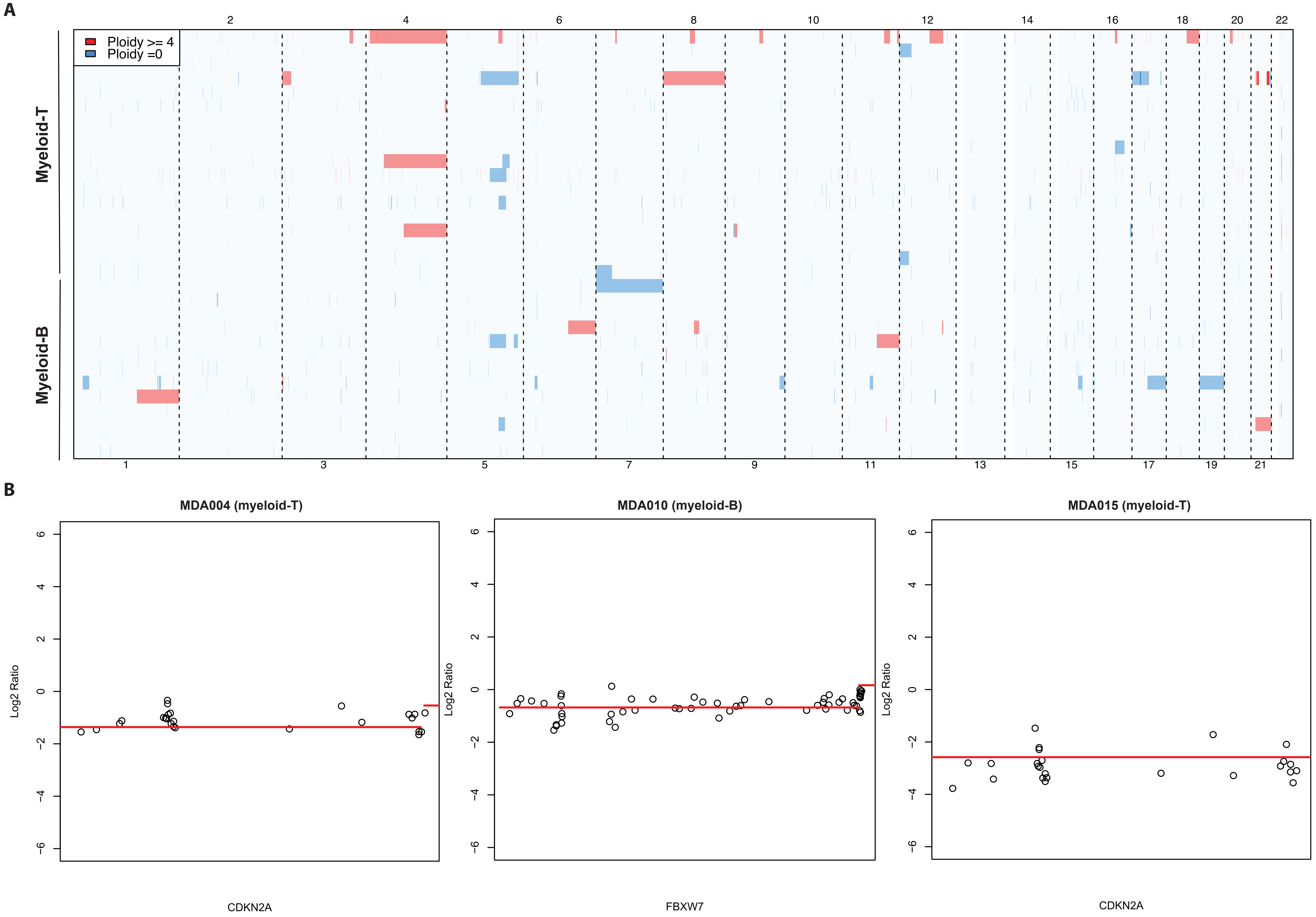

Supplementary Figure 4. (A) Genome wide copy number plot derived from methylation array in 31 MPAL samples. Arm level gain and loss were depicted by red and blue, respectively. **(B)** Some of the examples of focal copy number loss in selected genes.

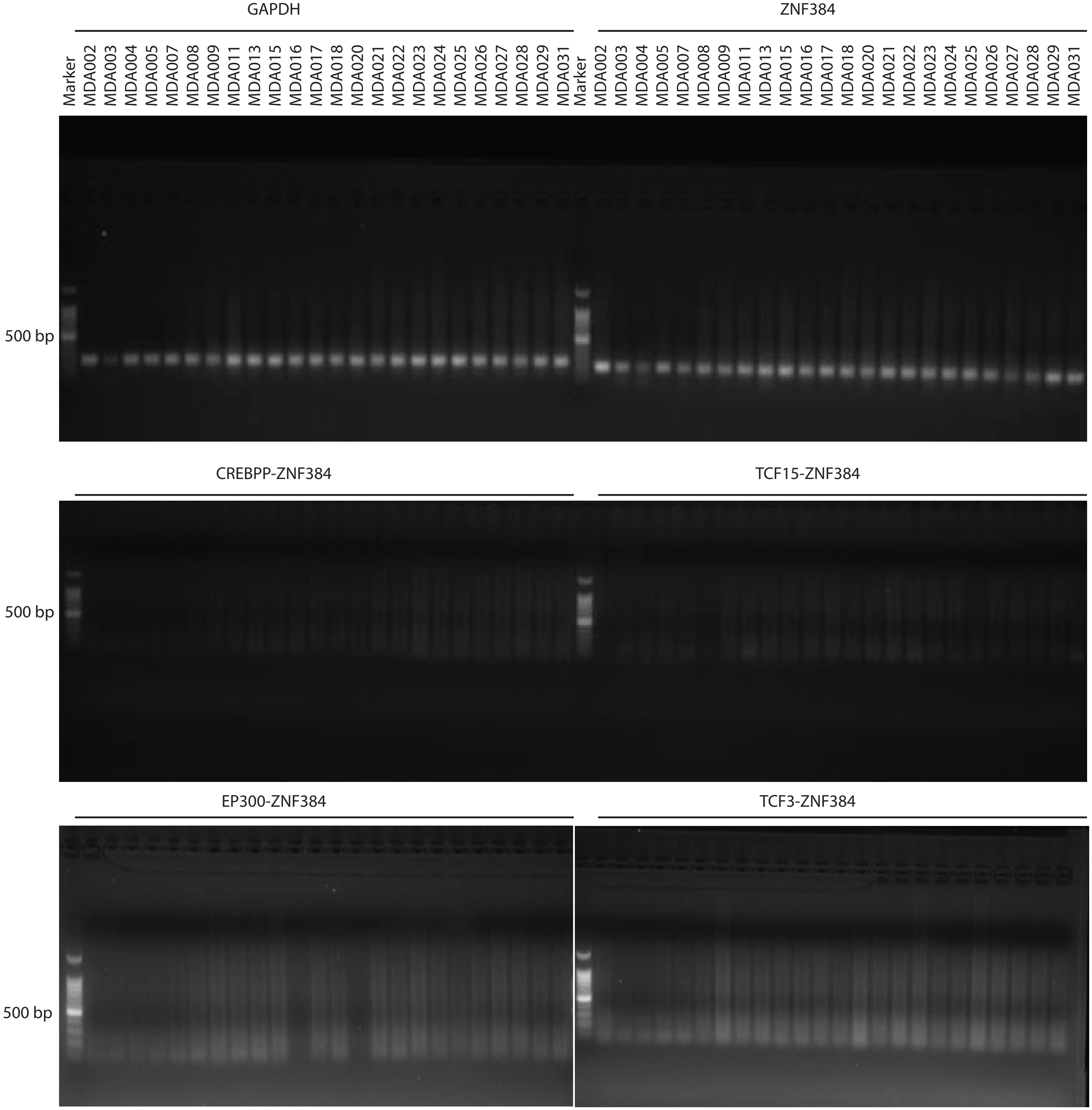

Supplementary Figure 5. Agarose gel electrophoresis of RT-PCR screening of most commonly reported ZNF384 fusions in 24 MPAL samples.Two positive control experiments were performed. First control produced 238 bp PCR bands of *GAPDH* transcript and second control produced 193 bp PCR bands of *ZNF384* transcript (exon 9-10) to confirm *ZNF384* expression in the samples. Details of PCR primers used in these experiments are summarized in Supplemnetal Method.

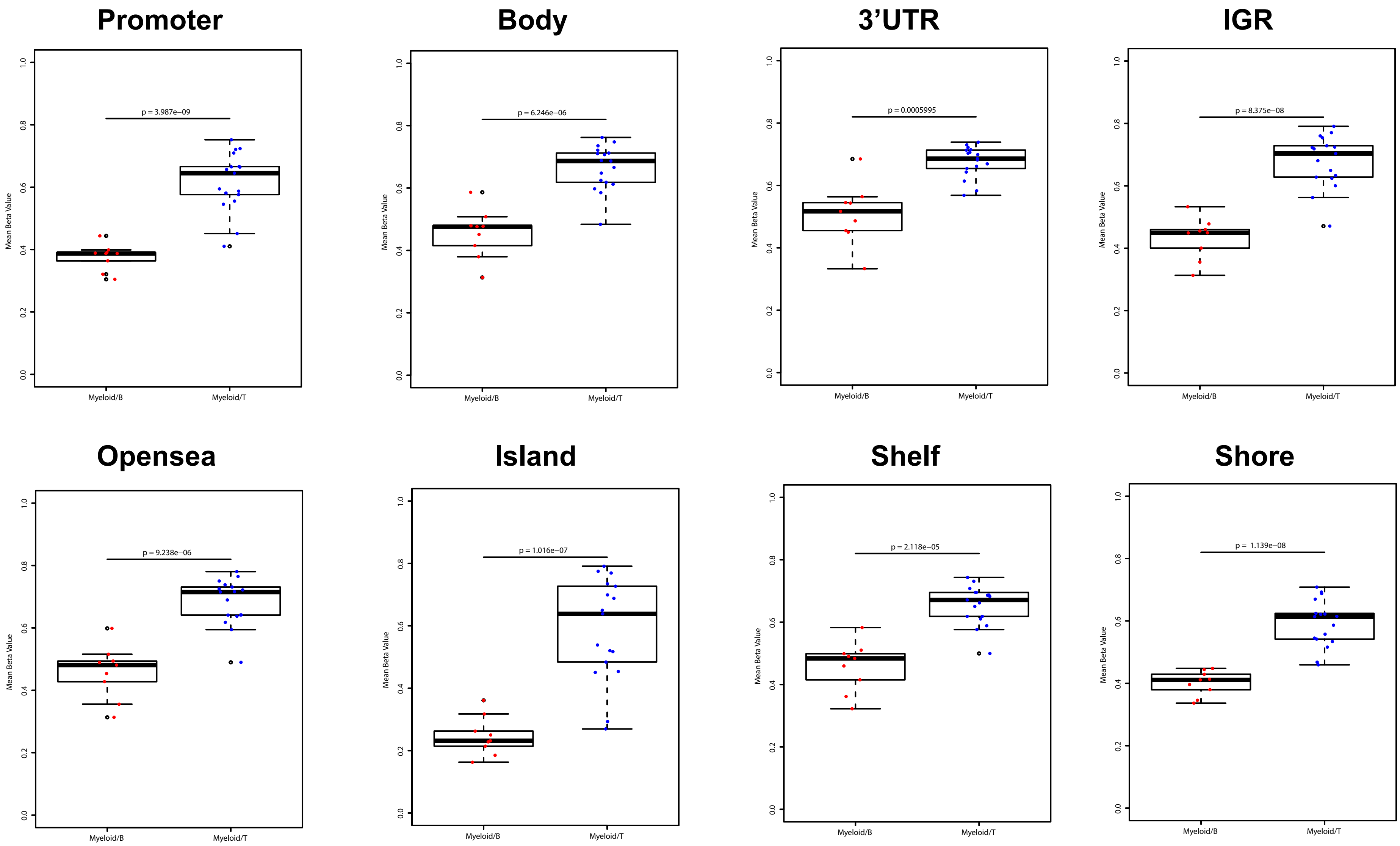

Supplementary Figure 6. Box plot comparing mean beta value of CpG probes based on the location.

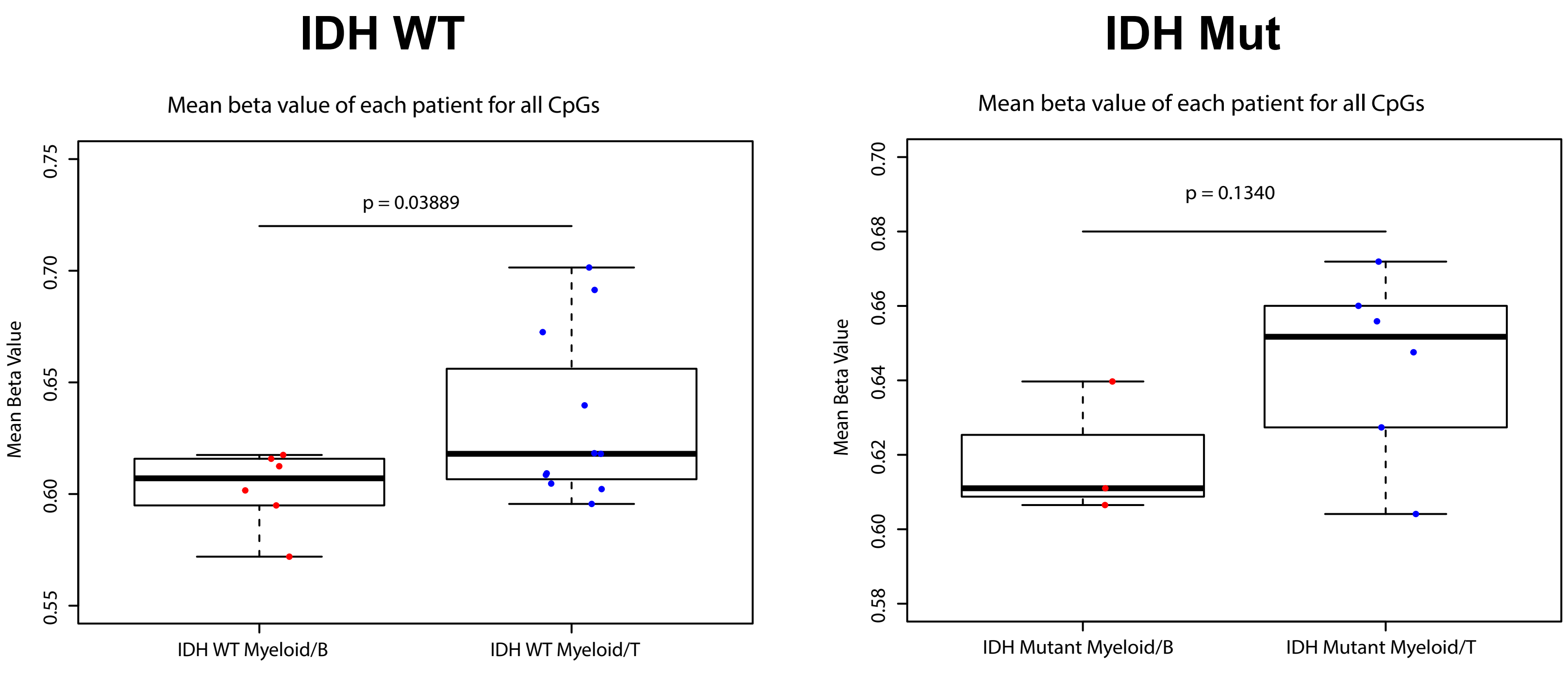

Supplementary Figure 7. Box plot comparing mean beta value of all CpGs between myeloid-B and myeloid-T MPAL based on IDH mutation status.

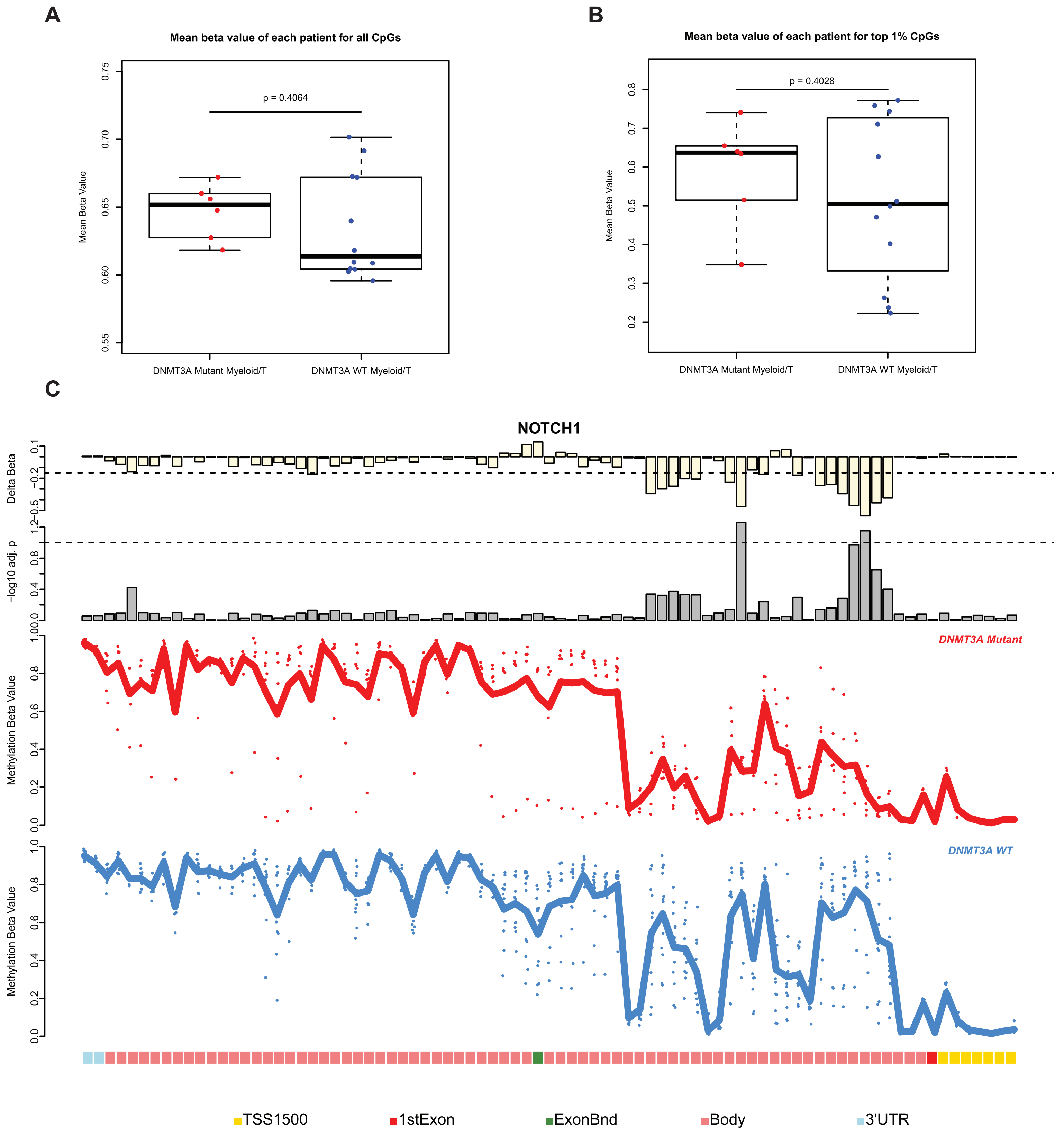

Supplementary Figure 8. Comparison of the overall methylation beta value between *DNMT3A* mutated and wild type (WT) cases in (**A**) all CpGs and (**B**) top 1% variable CpGs. Comparison is made among myeloid-T MPAL cases to avoid inherent methylation difference between myeloid-B and myeloid-T MPAL. (**C**) Difference in the methylated probes in *NOTCH1* gene by *DNMT3A* mutation. The top bar graph shows the difference in beta value at each loci by *DNMT3A* mutation. Dotted line shows delta beta value -0.15. The second bar graph shows inverted log adjusted P value of each loci. The lower two dot plots showing actual beta value at each loci in cases with *DNMT3A* mutated (red color) and WT (blue color) cases. The line shows the average.
The bottom shows the location of CpGs within the *NOTCH1* gene. Right is 5' and left is 3'.

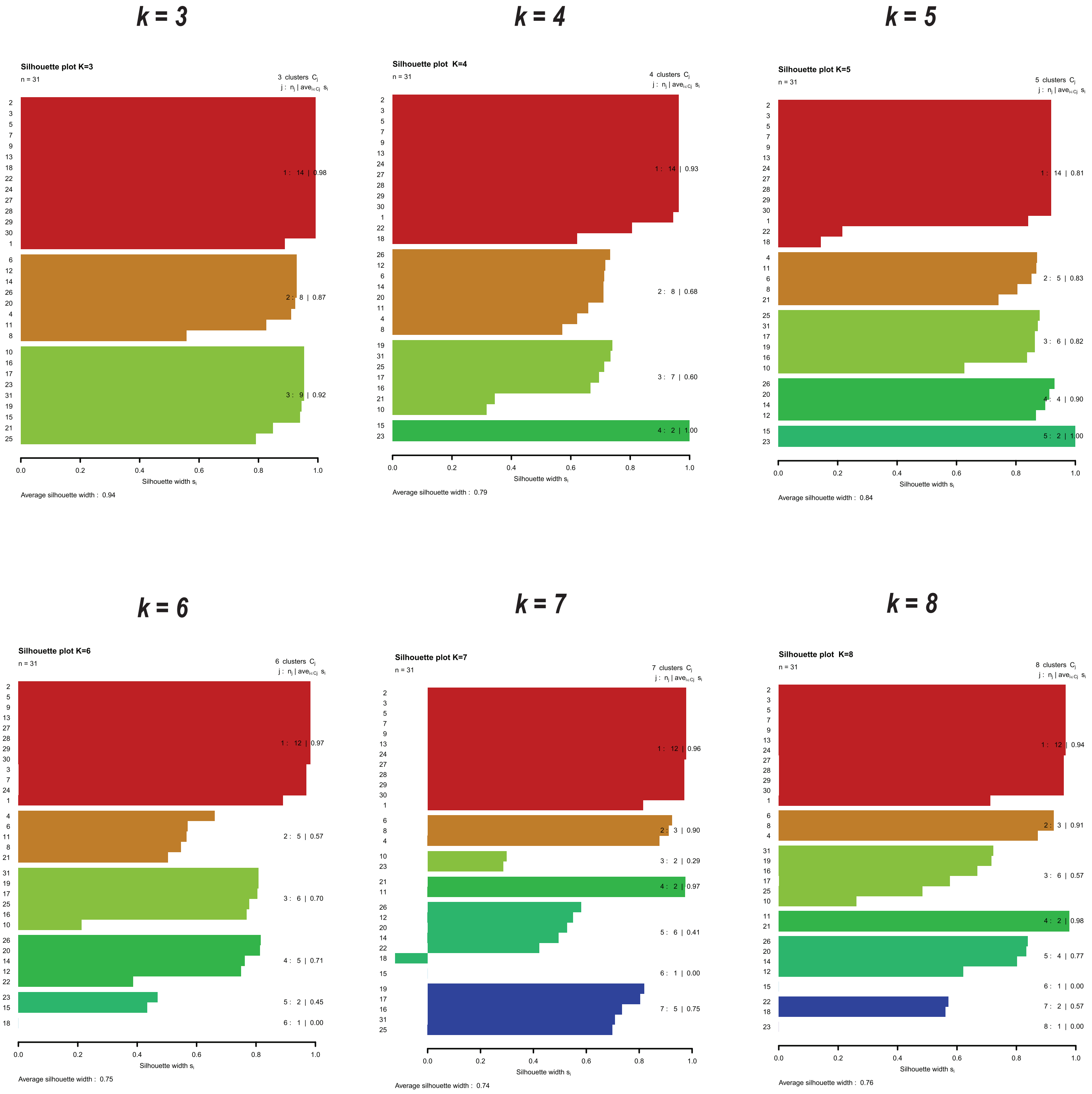

Supplementary Figure 9. Shilouette analysis for *k* means clustering on sample data with *k* = 3 to *k* = 8.
